# Small molecule mediated inhibition of protein cargo recognition by peroxisomal transport receptor PEX5 is toxic to *Trypanosoma*

**DOI:** 10.1038/s41598-022-18841-1

**Published:** 2022-08-29

**Authors:** Valeria Napolitano, Charlotte A. Softley, Artur Blat, Vishal C. Kalel, Kenji Schorpp, Till Siebenmorgen, Kamyar Hadian, Ralf Erdmann, Michael Sattler, Grzegorz M. Popowicz, Grzegorz Dubin

**Affiliations:** 1 Malopolska Centre of Biotechnology, Jagiellonian University, Gronostajowa 7a, 30-387 Kraków, Poland; 2 Faculty of Biochemistry, Biophysics and Biotechnology, Jagiellonian University, Gronostajowa 7, 30-387 Kraków, Poland; 3 Institute of Structural Biology, Helmholtz Zentrum München, Ingolstädter Landstraße 1, 85764 Neuherberg, Germany; 4 Bavarian NMR Center, Chemistry Department, Technical University of Munich, Garching, 85748 Munich, Germany; 5 Institute of Biochemistry and Pathobiochemistry, Department of Systems Biochemistry, Faculty of Medicine, Ruhr University Bochum, 44801 Bochum, Germany; 6 Institute of Molecular Toxicology and Pharmacology, Helmholtz Zentrum München, Ingolstädter Landstrasse 1, 85764 Neuherberg, Germany

**Keywords:** Infectious diseases, Biochemistry, Drug development

## Abstract

Trypanosomiases are life-threatening infections of humans and livestock, and novel effective therapeutic approaches are needed. *Trypanosoma* compartmentalize glycolysis into specialized organelles termed glycosomes. Most of the trypanosomal glycolytic enzymes harbor a peroxisomal targeting signal-1 (PTS1) which is recognized by the soluble receptor PEX5 to facilitate docking and translocation of the cargo into the glycosomal lumen. Given its pivotal role in the glycosomal protein import, the PEX5–PTS1 interaction represents a potential target to inhibit import of glycolytic enzymes and thus kill the parasite. We developed a fluorescence polarization (FP)-based assay for monitoring the PEX5–PTS1 interaction and performed a High Throughput Screening (HTS) campaign to identify small molecule inhibitors of the interaction. Six of the identified hits passed orthogonal selection criteria and were found to inhibit parasite growth in cell culture. Our results validate PEX5 as a target for small molecule inhibitors and provide scaffolds suitable for further pre-clinical development of novel trypanocidal compounds.

## Introduction

Trypanosomiases are severe, but still neglected diseases of human and animals caused by *Trypanosoma* species. *Trypanosoma cruzi* is the etiological agent of Chagas disease, also known as American Trypanosomiasis. Historically, Chagas disease was confined to rural areas of Latin America, but changes in lifestyle and large-scale population movements have increased the geographic distribution and changed the epidemiology. Nowadays, chronic Chagas disease creates a worldwide problem of high morbidity and substantial social impact^1^. It is estimated that worldwide about 6–8 million people are infected with *T. cruzi* and around 12,000 die because of the infection each year^2–4^.

*T. cruzi* infection spreads via bites of the triatomine bugs, known as kissing bugs. In non-endemic regions the transmission happens via non-vectoral routes including blood transfusion, organ transplantation or vertical transmission from mother to the child. The initial phase of the infection (known as an acute phase) can be asymptomatic or oligosymptomatic with fever, anorexia, and tachycardia^5^. In some cases, *Romaña*’s sign, a unilateral painless periorbital swelling, may manifest the acute phase. The acute phase lasts about 8–10 weeks upon which the infected individuals enter the lifelong chronic phase. In 60–70% cases the chronic phase is asymptomatic (a state known as indeterminate Chagas disease). The remaining 30–40% of chronically infected individuals gradually progress to an advanced chronic phase with neurological, cardiac, digestive (megacolon or megaesophagus), or cardio-digestive clinical manifestations which can appear even 10–30 years after acute infection^1,2,6^. Cardiac involvement is the most frequent and severe complication and occurs in 14–45% of chronically infected patients. Heart failure (70%) and sudden death (30%) are the most common causes of mortality in subjects with Chagas disease^7^.

Benznidazole and nifurtimox are the only drugs available to treat Chagas disease. Both drugs have been the mainstay of antitrypanosomal treatment for almost 50 years, although their safety and efficacy profiles are far from ideal. The drugs are recommended in acute stage or in the chronic stage when asymptomatic and without organ implications. The drugs cannot be administrated during pregnancy and in patients with hepatic or kidney disease. Nifurtimox is also contraindicated in neurological or psychiatric disorders^8^. The biggest problem however, is that while the efficacy in the acute stage of the disease ranges from 70 to 80%, it drops dramatically to 6–10% cure rate in the chronic stage^8^. Furthermore, adverse effects of a required long-term treatment frequently lead to discontinuation and treatment failure.

Novel, effective drugs are desperately needed, but the clinical pipeline is very thin. Fexinidazole, a 5-nitroimidazole derivative that inhibits DNA synthesis, was developed by the Drugs for Neglected Diseases initiative (DNDi), in collaboration with Sanofi, for the oral treatment of Human African Trypanosomiasis (HAT). A clinical study is currently ongoing in Spain to evaluate the efficacy and safety of short-course and low-dose regimens in adults with chronic Chagas disease^9^, but if the study is unsuccessful, alternatives are few and only at preclinical stages.

Glycosomes are peroxisome-like organelles compartmentalizing metabolic processes and were identified as valid targets in trypanosomiasis^10–12^. The hallmark of *Trypanosoma* is that glycosomes sequester the majority of the glycolytic enzymes. It has been demonstrated that erroneous glycosome function leads to metabolic catastrophe and death of the parasites^13^. Peroxins (PEX) are essential for biogenesis of glycosomes and glycolytic enzyme import. In particular, PEX5 receptor is responsible for cargo recognition and docking to the glycosomal membrane. Docking of the PEX5–cargo complex at PEX14 results in formation of a dynamic transient import pore which allows translocation of cargo into the glycosomal lumen^14^. Dawidowski et al*.* demonstrated that inhibition of PEX5–PEX14 interaction with small molecules disrupts glycosomal protein import and kills the parasites both in vitro and in vivo^12^*.* Same effect could be expected for the disruption of PEX5–cargo recognition while PEX5 would allow for different chemistries compared to PEX14.

**Figure 1:**
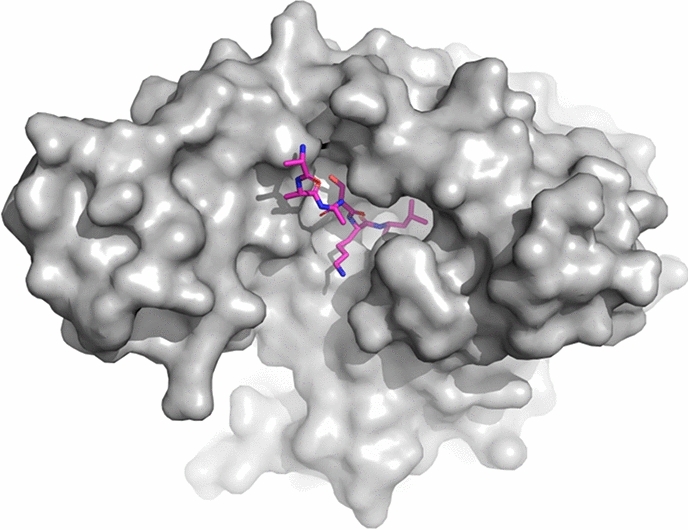
PTS1 binding pocket of PEX5 as a potential target for inhibitor development. Surface representation of *Trypanosoma brucei* PEX5 (grey) in complex with PTS1 (magenta sticks) (PDB code: 3CVP). Structure of *T. cruzi* PEX5 is not available, but high sequence identity of *T. cruzi* and *T. brucei* Pex5 allows to expect similar binding mode.

The C-terminal tetratricopeptide repeat (TPR) domain of PEX5 recognizes peroxisomal targeting signal 1 (PTS1). The PTS1 is a tripeptide with a consensus Ser-Lys-Leu sequence at the C-terminal of cargo proteins^15^. PTS1 locates in a deep groove within TPR contributing a significant number of hydrogen bonds^16,17^. The deep PTS1 binding pocket of TPR (Fig. 1) seems convenient for inhibitor discovery. Nonetheless, PEX5 still awaits validation as a target.

This study identifies small molecule inhibitors of PEX5 interaction with the cargo and validates PEX5 as a relevant target in trypanosomiasis paving the road to future development of pharmacologically relevant small molecules.

## Results

**Figure 2:**
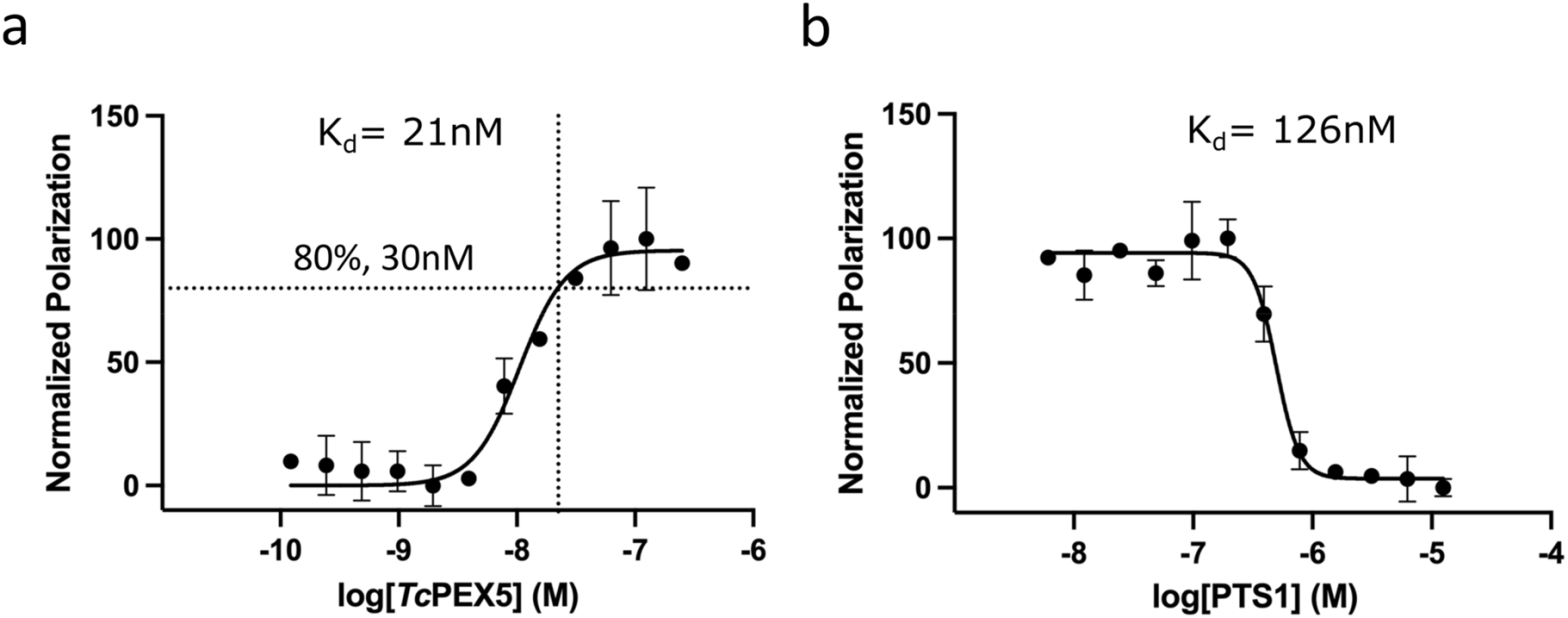
Fluorescence polarization (FP) based PEX5–cargo interaction assay. (**a**) PTS1 (10 nM, 6-FAM labeled) was titrated with *Tc*PEX5 (TPR domain) and changes in the polarization of emitted light were monitored. Affinity (K_d_) was determined by curve fitting. f_0_ point was determined at 30 nM *Tc*PEX5. (**b**) Competitive FP assay—increasing concentrations of unlabeled PTS1 were added to 30 nM *Tc*Pex5 (f_0_) and 10 nM labeled PTS1 mixture and the changes in the polarization of emitted fluorescence were monitored. K_i_ was determined by curve fitting. (**a**,**b**) Each data point represents an average of three independent measurements with the standard deviation indicated. Polarization values were normalized by setting the average FP readout for the free FAM-PTS1 at 0% and the average FP value for PEX5 saturated FAM-PTS1 at 100%.

To identify PEX5–cargo interaction inhibitors, an assay was developed allowing to monitor the interaction of peroxisomal targeting sequence 1 (PTS1) and the C-terminal tetratricopeptide-repeat (TPR) domain of PEX5. A pentapeptide representing PTS1 was fluorescently labeled at the N-terminus by conjugation with 6-carboxyfluorescein (6-FAM). The C-terminal TPR domain of PEX5 was expressed in heterologous system and purified to homogeneity. The interaction of labeled PTS1 and PEX5 was monitored by fluorescence polarization. Affinity (K_d_) of 6-FAM-PTS1 for PEX5 was determined at 21 nM by direct titration (Fig. 2a), a value in rough agreement with that reported previously (70 nM)^16^.

A displacement assay was used for detection of active compounds. The concentration of the labeled probe (10 nM) was set below the K_d_ value and the concentration of PEX5 (~ 30 nM) was adjusted to obtain 80% saturation (f_0_), a tradeoff between reasonable dynamic range (~ 100 mP) and sensitivity^18^. The displacement was validated using unlabeled PTS1 peptide as a competitor. In such conditions the K_i_ characterizing the unlabeled PTS1 peptide was determined at 126 nM (Fig. 2b). This data demonstrates that although the bulky florescent label provides additional points of contact as evidenced by increased affinity compared to PTS1 alone, the displacement assay is still suitable for detecting competitive inhibitors of PTS1–PEX5 interaction.

The assay was miniaturized to 384 well plate format and its suitability for high-throughput screening was evaluated by Z′ test, a statistical parameter assessing both the assay dynamic range and the data variation^19^. The Z′ factor was consistently above 0.8 in all the tests indicating excellent screening quality of the test. When the effect of DMSO (solvent used for small molecule handling) was evaluated, it did not affect the Z′ up to 5% *v/v* concentration. No higher DMSO concentration was used in this study in screening or K_i_ assessment.

The FP-based displacement assay was used to screen an in-house library of ~ 30,000 compounds for PEX5–PTS1 interaction inhibitors. A cut-off value for active compounds was set at 4 times the standard deviation below median of all compound-treated wells (calculated per plate). Hits representing auto-fluorescent compounds were excluded based on total fluorescence. Frequent hitters were excluded based on results of prior screening campaigns. The remaining 48 compounds were considered as valid HTS hits.

**Figure 3:**
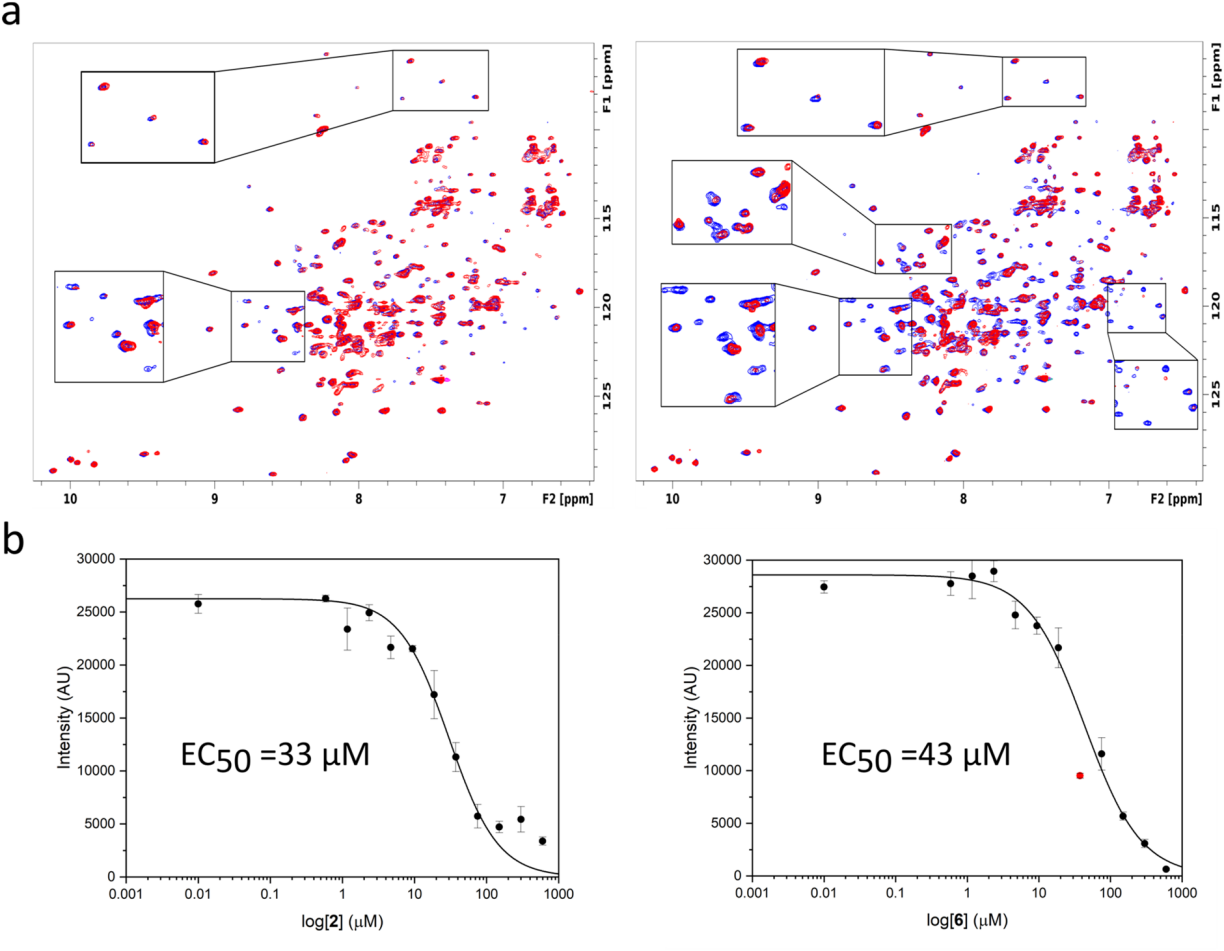
Orthogonal validation of HTS hits. (**a**) ^1^H–^15^N 2D HSQC spectra of the ^2^H, ^15^N-labeled *Tc*PEX5 (blue) superimposed with spectrum after addition of hit compound **2** (left) and **6** (right) at 1:1 molar ratio (red). Zoomed views show significant spectral changes. (**b**) AlphaScreen dose-dependent displacement curves for **2** (left) and **6** (right). The error bars represent the standard error of n = 4 measurements.

HTS hits were validated using protein-detected NMR experiments. Recombinant ^2^H, ^15^N labeled TPR domain of PEX5 was obtained by expression in minimal media containing ^15^NH_4_Cl as the sole nitrogen source in D_2_O. ^1^H, ^15^N HSQC (heteronuclear single quantum coherence) NMR spectra were used to monitor amide chemical shift changes and/or line-broadening during titrations with the best HTS hit compounds. Significant spectral changes at 1:1 compound to PEX5 molar ratio were observed for six compounds (Table 1; Fig. 3; Fig. S1).

**Table 1:** Chemical structures, EC_50_ values (AlphaScreen) and trypanocidal activity of hit compounds identified in this study.

#	Chemical structure	*Tc*PEX5-PTS1 EC_50_ [µM] (AlphaScreen)	*T. brucei brucei* IC_50_ [µM]^a^
1		190 ± 37.6	2.18 (1.63–2.89)
2		33.0 ± 3.1	12.4 (9.52–15.8)
3		705 ± 85.2	3.84 (1.70–8.33)
4		78.2 ± 7.4	6.45 (3.10–13.6)
5		411 ± 98.2	3.84 (1.14–11.4)
6		43.4 ± 6.3	6.89 (5.59–8.27)

^a^EC_50_ values are shown as mean (n = 4). Values in parentheses are 95% confidence intervals.

The six compounds which passed the NMR titration selection criterion were purchased from commercial vendors with certificates of analysis. As a second orthogonal selection criterion, EC_50_ for each compound was determined by AlphaScreen monitored titration. To this end, the dissociation of the interaction of N-His-PEX5 and biotinylated PTS1 by tested compounds was monitored by principles of AlphaScreen proximity assay. Hit compounds **2** and **6** were characterized by highest affinity (Fig. 3b, Table 1), which correlated with NMR analysis in which the compounds were characterized by most significant chemical shift perturbations in ^1^H–^15^N-HSQC experiments (Fig. 3a). Hits **4** and **6** are especially interesting given their relatively high affinity at relatively low molecular weight compared to other identified compounds.

**Figure 4:**
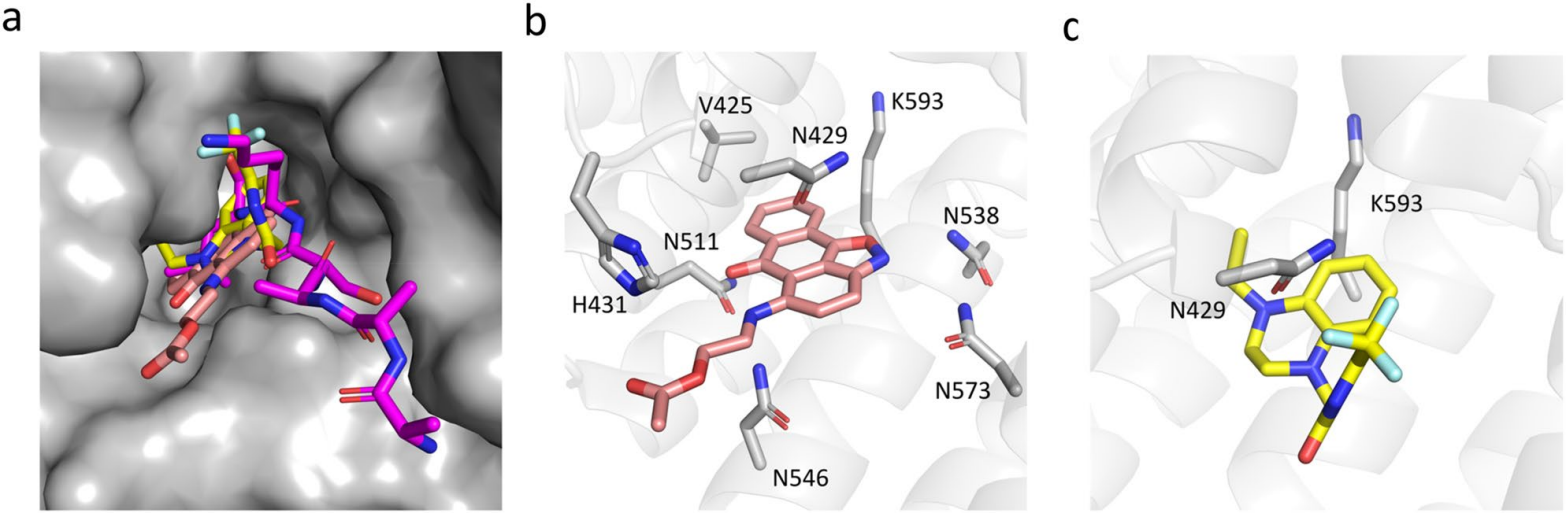
Putative binding modes (docking poses) of compounds **2** and **6** at the PTS1 binding pocket of PEX5. (**a**) Surface representation of the PTS1 binding site (grey). Compounds **2** (pink) and **6** (yellow) overlay with the SKL motif of the PTS1 (magenta—PDB code:3CVP) (**b**,**c**) docking poses of compounds **2** and **6**, respectively. Residues important for binding are highlighted as sticks.

Given the diversity of chemical structures of the identified compounds we asked if they all bind at the PTS1 pocket of PEX5 displacing the cargo, or possibly some acted as allosteric modulators. Induction of comparable chemical shifts by compounds **2** and **6** suggests similar binding site, but the small extent of observed chemical shifts and lack of assignment did not allow to obtain conclusive answer. We have thus turned to computational prediction of the putative binding sites and docked all the compounds at the surface of PEX5. The docking simulations gave convincing predictions of the binding poses of compounds **2** and **6**. The overall binding of **2** and **6** resembles the interaction of PTS1 at the central cavity of the TPR domain of PEX5 (Fig. 4a). A number of asparagine residues are involved in PEX5 recognition of PTS1. With some rearrangement, Asn 511, Asn 546 and Asn 573 could form favorable interactions with compound **2**. Moreover, CH–π interactions between the aromatic rings of compounds **2** and **6** and the side chain of Lys 593 and Val 425 (only for compound **2**) further support the binding (Fig. 4b,c).

Having demonstrated a direct interaction of each hit compound and the target protein by 2D NMR and having obtained a reasonably shaped dose response curves in a dissociation assay using compounds of confirmed identity, we considered hits **1**–**6** as sufficiently verified to progress to validation in parasites. *T. brucei brucei* bloodstream form parasite was used as a model organism and the effect of hit compounds on the viability of the parasites was evaluated in the resazurin-based cell survival assay. All tested compounds were effective against *T. b. brucei* in the assay conditions and were characterized by low micromolar trypanocidal activity (IC_50_) demonstrating the potential of PEX5 inhibition as a strategy against *Trypanosoma*.

## Discussion

Peroxisomal protein transport is vital to *Trypanosoma* and the PEX system was demonstrated as a valid target in Trypanosomiases. Effective transport via PEX system involves more than a dozen components, and it remains to be determined which are best targeted to enable efficient drug design, efficacy and safety. Targeting the PEX14–PEX5 protein–protein interaction, which is essential for glycosomal protein import, was recently introduced as a novel concept to develop trypanocidal inhibitors and proved effective in killing *Trypanosoma*^12^. However, the hydrophobic protein–protein interaction surface is challenging for medicinal chemistry optimization. Here we report identification of a number of scaffolds capable of inhibiting the cargo interaction with PEX5, a receptor offering a spacious and deep pocket ideal for drug design. We demonstrate that the identified hits bind directly to PEX5 protein in a dose dependent manner.

We further demonstrate that the identified hit compounds effectively kill *T. brucei* parasites in the cell culture. *T. brucei* is related to *T. cruzi* (the cause of Chagas disease) and was used here as a model because of lesser safety concerns. PEX proteins and PTS-containing glycosomal enzymes are highly conserved between the two species. Previously it was shown that the small molecule inhibitors developed against *T. brucei* PEX14–PEX5 interaction could also kill *T. cruzi* parasites^12^, justifying the use of *T. brucei* as model organism in the initial phase of inhibitor development.

The trypanocidal activity of identified compounds (IC_50_) exhibits different trend compared to potency in dissociating the PEX5–PTS1 complex in vitro (EC_50_). This, however, may be explained by the diversity of chemical structures of identified compounds and the complexity of the killing assay which utilizes the whole organisms. Unlike the activity in dissociating purified proteins in the test tube (Alphascreen), the trypanocidal activity is influenced by pharmacokinetics (membrane permeability, stability, metabolism and a variety of other factors) which was not considered in this study. It is evident from the difference of EC_50_ and IC_50_ that pharmacokinetics has an important role in limiting the activity of identified compounds. Finally, at the current stage we cannot exclude that some of the compounds exhibit off target effects. Further study analysing the Structure Activity Relationship (SAR) will provide a better explanation. Despite the unknowns, however, our study allowed to identify PEX5 inhibitors capable of penetrating the cell membrane, dissociating PEX5–PTS1 interaction and killing the parasite, while further investigation is necessary to optimize the compounds.

Our results provide the first direct evidence that it is possible to interfere with PEX5–cargo interaction by using small molecules and that PEX5 inhibition is toxic to *Trypanosoma*. Therefore, PEX5 inhibitors may find use in antiparasitic drug development. Nevertheless, the presented compounds need optimization for better affinity and their effect against *T. cruzi* requires experimental evaluation. Furthermore, the efficacy of optimized compounds in animal models needs to be validated. Our study suggests that inhibition of PEX5 is a valid target in *Trypanosoma*. Further studies will focus on optimizing the identified hits towards trypanocidal drugs.

## Methods

### Protein expression and purification

Gene encoding the TPR domain of *T. cruzi* PEX5 (347–668) was optimized for *E. coli* codon usage, synthesized by Integrated DNA Technologies (Coralville, USA) and cloned between NcoI and NotI restriction enzyme sites into petHSU vector. The resulting construct contained an N-terminal Hexa-histidine (His_6_) tag and a SUMO tag. For AlphaScreen the same gene was amplified using forward 5′-TACGACCATATGGAAACCAATTATCCTTTTG and reverse 5′-TACGACCTC GAGAACCGCCATGTCCTCCAAG primers and cloned into pET-24a(+) vector between NdeI and XhoI restriction sites resulting in a construct containing C-terminal His_6_-tag.

The relevant plasmid was transformed into *E. coli* BL21 (DE3). A single colony was inoculated in 50 mL LB medium containing 10 µg/mL kanamycin and incubated overnight at 37 °C. 5 mL of the preliminary culture were used to inoculate 500 mL of LB medium supplemented with 50 µg/mL kanamycin and incubated at 37 °C. When the OD_600_ reached 0.8, the culture was cooled to 20 °C, induced with 1 mM isopropyl β-d-1-thiogalactopyranoside (IPTG) and the culture was continued overnight. The cells were then harvested by centrifugation and resuspended in lysis buffer containing 50 mM Hepes pH 7.5, 300 mM NaCl, 20 mM imidazole, 10 mM β-mercaptoethanol, 40 µM AEBSF-HCL (protease inhibitor), 1 µg/mL DNAaseI and lysed on ice by sonication. The lysate was clarified by ultracentrifugation. The supernatant was applied to a HiTrap IMAC column pre-equilibrated with the lysis buffer and washed with abundant washing buffer (50 mM Hepes pH 7.5, 300 mM NaCl, 20 mM imidazole, 10 mM β-mercaptoethanol). The His-SUMO tag was cleaved off overnight, directly on column, using dtUD1 protease. The flow-through was then collected, concentrated to 5 mL using a 30 kDa cutoff Amicon Ultra filter and applied to a size exclusion chromatography on High load S75 pre-equilibrated with 20 mM Hepes pH 7.5, 100 mM NaCl and 5 mM β-mercaptoethanol. 6-His-tagged *Tc*PEX5 variant was concentrated to 5 mL and further purified by size exclusion chromatography on High load S75 pre-equilibrated with PBS supplemented with β-mercaptoethanol straight after being eluted from HiTrap IMAC column. The protocol yielded on average 30 mg of *Tc*PEX5 from 1 L of bacterial culture.

Perdeuterated ^15^N-labelled *Tc*PEX5 was expressed in M9 minimal medium prepared in D_2_O and containing ^15^N-ammoniun chloride as the sole nitrogen source. 5 mL of preliminary culture were used to inoculate 500 mL of the same medium. Cells were grown at 37 °C until the OD_600_ reached 0.8, the culture was cooled to 18 °C, induced with 1 mM IPTG and maintained overnight. The pellets were collected and the protein was purified as described above. In the last step of purification, the protein was applied to a size exclusion chromatography on S75 pre-equilibrated with NMR buffer (50 mM phosphate buffer pH 7.4, 150 mM NaCl and 5 mM β-mercaptoethanol).

### Fluorescence polarization (FP) binding assay

All FP measurements were performed on a multifunctional microplate reader (Tecan InfinitePro F200 plate) in Corning NBS black 96-well or 384-well NBS microplates. 485-nm excitation and 535-nm emission filters were used. The FP values were calculated as follows:

$$FP=\frac{{I}_{\parallel }-{I}_{\perp }}{{I}_{\parallel }+ {I}_{\perp }}$$

where ${I}_{\parallel }$ and ${I}_{\perp }$ are the emission light intensity parallel and perpendicular to the excitation light plane, respectively. Fluorescence polarization values were expressed in millipolarization units (mP).

In the FP saturation binding experiment, 10 nM 6-FAM-labelled PTS1 (6-FAM-YQSKL) was mixed with increasing concentrations of *Tc*PEX5 (0.1–250 nM) in FP buffer containing 10 mM Hepes pH 7.5, 100 mM NaCl and 5% DMSO. Each data point was determined in triplicate. The FP values were plotted against the log_10_ of the protein concentration, and the dissociation constant (K_d_) was obtained by fitting the experimental data using an equation representing a one site non-cooperative ligand binding:

$$FP={FP}_{min}\times \frac{\left({FP}_{max}- {FP}_{min}\right)\times c}{{K}_{d}+c}$$

where FP is the determined value of the fluorescence polarization, FP_min_ is the value of the fluorescence polarization of the peptide alone, FP_max_ is the maximum value of the fluorescence polarization (saturation), K_d_ is the dissociation constant and c is the protein concentration.

Competitive binding experiment was performed at 10 nM 6-FAM-labeled PTS1 and *Tc*PEX5 concentration yielding f_0_ = 0.8 according to Huang^18^. An in-house 30,000 molecules diversity set (ChemBridge, ChemDiv, Enamine, PPI) and FDA-approved drug library were used in high throughput screening. The selection criteria for the diversity libraries are: (i) diversity within each library and between the 3 diversity sets; (ii) MW < 600 g/mol; (iii) compounds with acceptable logS/logP for solubility; (iv) Lipinski’s rule of 5; (v) Purity > 90%. Reactive, unstable and toxic chemical groups, chemotypes of known acute or chronic toxicity and trivial compounds present in commercial random libraries have been filtered out. Each tested compound (50 mM in DMSO) was transferred into each well of 384-well assay plate with a robotic delivery system. Mixtures containing 30 nM *Tc*PEX5 and 10 nM 6-FAM-PTS1 were dispensed into the compound containing wells with a reagent dispenser. In each assay plate, DMSO and unlabeled PTS peptide were used as negative and positive controls, respectively. Wells containing 10 nM 6-FAM-PTS1 only were used as additional controls. The inhibitory activities were calculated using the following equation: %Inhibition = 100 × (mPn − mPs)/(mPn − mPp); where mPn, mPp, and mPs represent FP values of the negative controls, positive controls, and compound samples, respectively.

Prior to HTS the assay performance was evaluated using Z′ test according to^19^:

$${Z}^{{\prime}}=\frac{1-(3{SD}_{n}+3 {SD}_{p})}{{\mu }_{n}-{\mu }_{p}}$$

where SD_n_ and SD_p_ are the standard deviations, and μ_n_ and μ_p_ represent the means of the FP values obtained from the negative and positive controls, respectively. For this test each 384-well plate contained 190 negative control wells (labelled peptide and protein), 190 positive control wells (labeled peptide, protein, and unlabeled PTS1), and four 6-FAM-PTS1 only wells. All experiments were repeated three times.

### NMR spectroscopy

^1^H, ^15^N heteronuclear single quantum coherence (HSQC) spectra were measured for uniformly perdeuterated ^15^N-labeled *Tc*PEX5 (120 µM) in the absence or presence of ligands at 1:1 protein:ligand molar ratio. 10% (v/v) of D_2_O was added to the samples to provide the lock signal. Water suppression was carried out using the WATERGATE sequence^20^. All spectra were recorded at 298 K using a Bruker Avance 600 MHz spectrometer with a cryogenic TCI probehead. ^1^H–^15^N heteronuclear correlations were obtained using the SOFAST-HSQC experiment^21^. Spectra were processed and visualized using TopSpin 4.0.2.

### AlphaScreen assay

AlphaScreen assay was used as an orthogonal assay to test the ability of compounds of interest to dissociate PEX5–PTS1 interaction. 100 nM N-His-PEX5 was mixed with 50 nM biotinylated PTS1 (YQSKL) in a PBS buffer supplemented with 5 mg/mL of BSA and 0.01% (v/v) Tween-20. 5 µg/mL of streptavidin donor beads and 5 µg/mL of nickel chelate acceptor beads (PerkinElmer) were added to the mixture. For EC_50_ determination, serial dilutions of the inhibitors prepared in DMSO were added while keeping constant concentration of DMSO at 5% (this concentration was shown to have no effect on the assay readout). Signal was determined according to the bead manufacturer instructions. Data were analyzed using Origin Pro 9.0. Experimental points were interpreted using Hill sigmoidal fitting fixing the asymptotes at the maximal assay signal (no inhibitor added) and 0, respectively.

### Trypanocidal activity assay

The trypanocidal activity of tested compounds was evaluated against *T. brucei brucei* bloodstream form (BSF) using resazurin-based 96-well plate assay. *T. b. brucei* BSF (Lister 427, MITat 1.2) parasites were grown in HMI-11 medium containing 10% fetal bovine serum (FBS) at 37 °C at 5% CO_2_. 1:1 serial dilutions (10 points) were prepared in quadruplicates for each compound in HMI-11 medium (100 µL/well). Additionally, each row contained a well without a compound and one with medium solely as controls. 100 μL of parasite cultures (4 × 10^3^/mL) were inoculated in all wells (except the control with medium alone) so that the final concentration of parasites was 2 × 10^3^/mL. The plates were incubated for 66 h. 25 µL of 0.1 mg/mL resazurin (in Hanks Balanced Salt Solution) was added to each well and further incubated till 72 h timepoint. The reduction of resazurin was detected by following the fluorescence emission at 585 nm (excitation 530 nm) using a Synergy H1 microplate reader. The fluorescence emission of the well containing medium only was considered as background and subtracted from the fluorescence emission of other wells; then the percent survival values were calculated setting the fluorescence emission of the well without the compound at “100% survival”. Experimental data points were fitted with a non-linear regression using GraphPad (6.04) and the half-maximal inhibitory concentration (IC_50_) values were derived from the corresponding sigmoidal dose–response curves.

## Supplementary Information

Supplementary Figure S1.

## Data Availability

The authors declare that the data supporting the findings of this study are available within the paper.
