# Supplementary material for: Small molecule mediated inhibition of protein cargo recognition by peroxisomal transport receptor PEX5 is toxic to Trypanosoma
Source: Sci Rep. 2022 Aug 29;12:14705. doi: 10.1038/s41598-022-18841-1 (PMC9424529; doi:10.1038/s41598-022-18841-1)
Supplement: Supplementary file 1 — Supplementary Figure S1. [file 41598_2022_18841_MOESM1_ESM.docx]

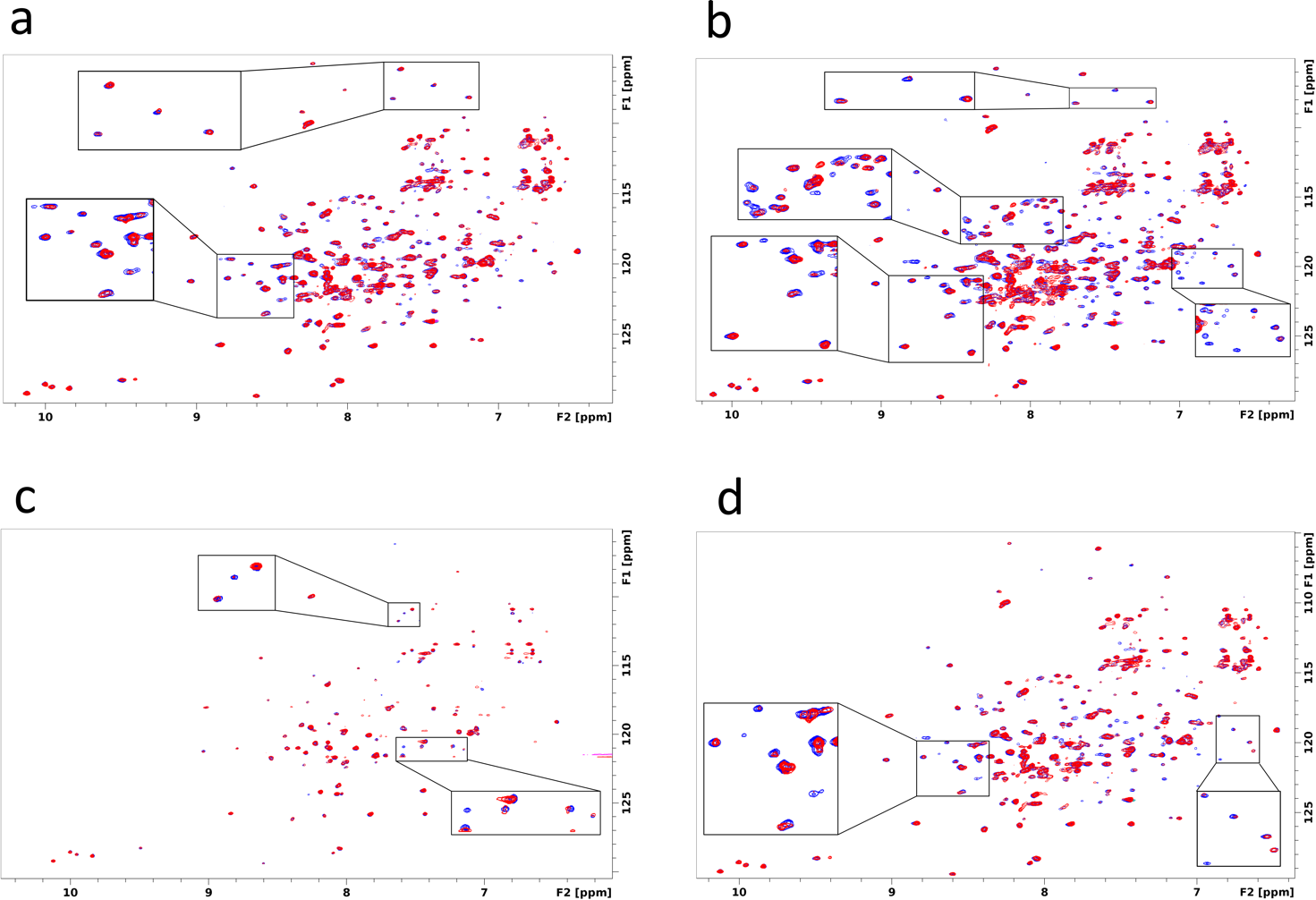


**Figure S1.** **NMR spectra.** ^1^H-^15^N 2D HSQC spectra of the ^2^H,^15^N-labeled TcPEX5 (blue) superimposed with spectra after addition of hit compound (a) 1, (b) 3, (c) 4 and (d) 5 at 1:1 molar ratio (red). Regions of significant difference are highlighted.
